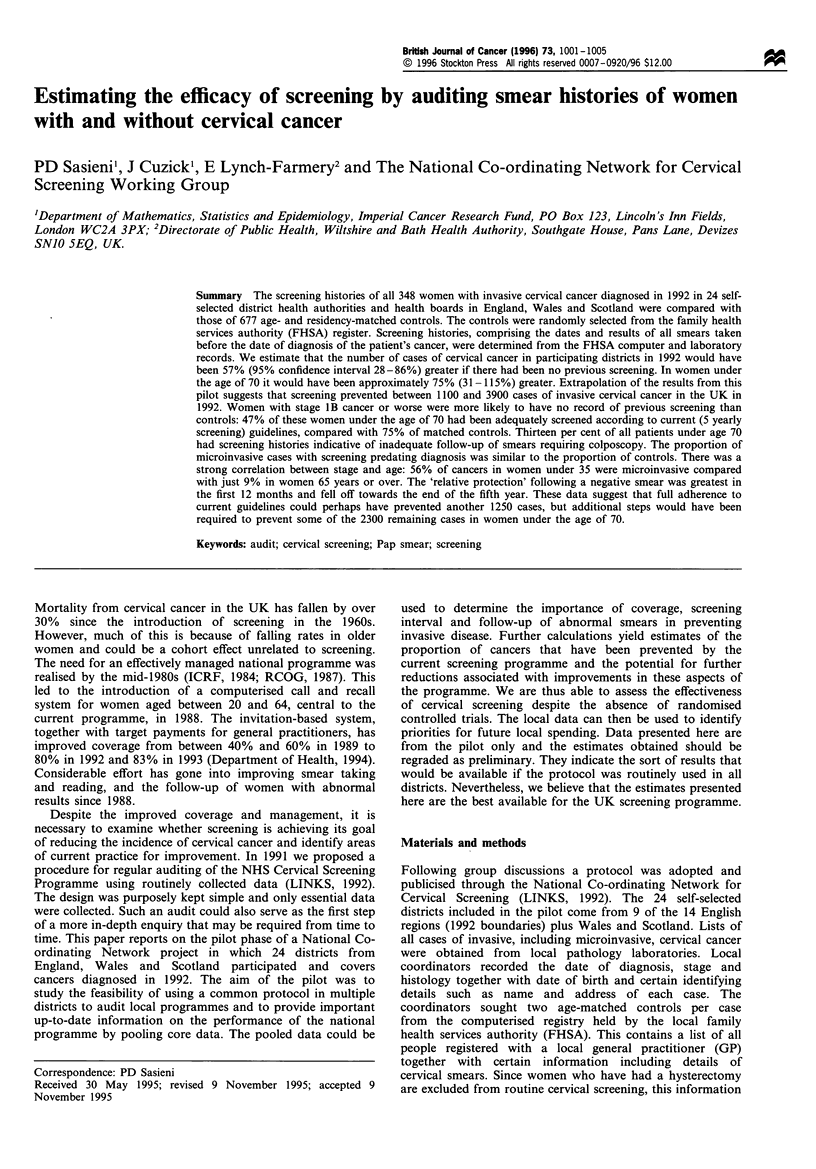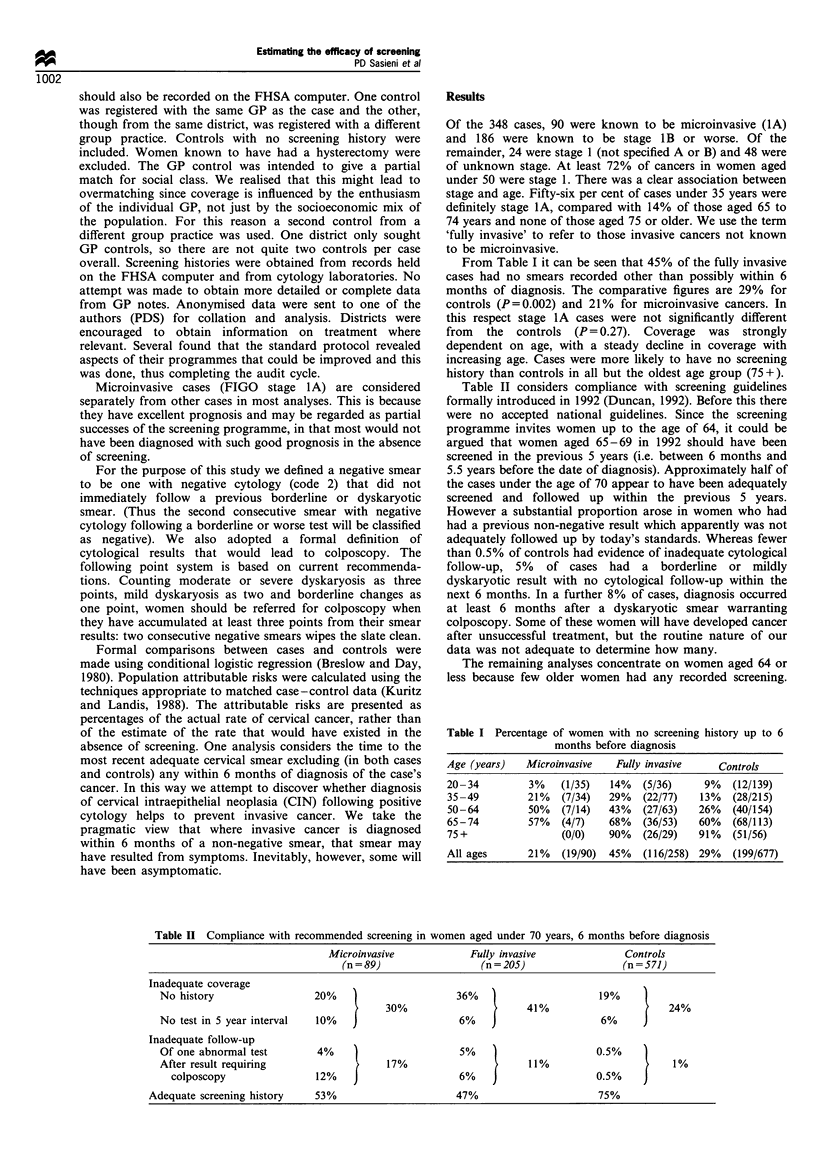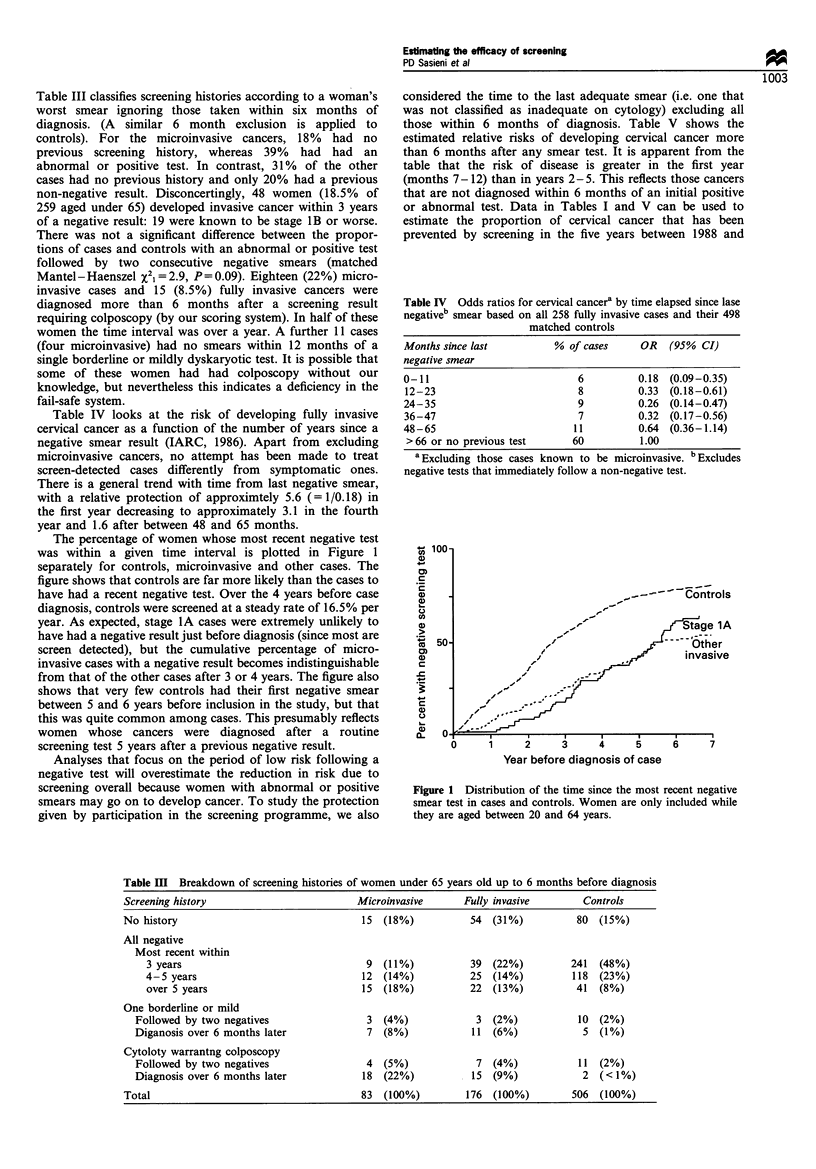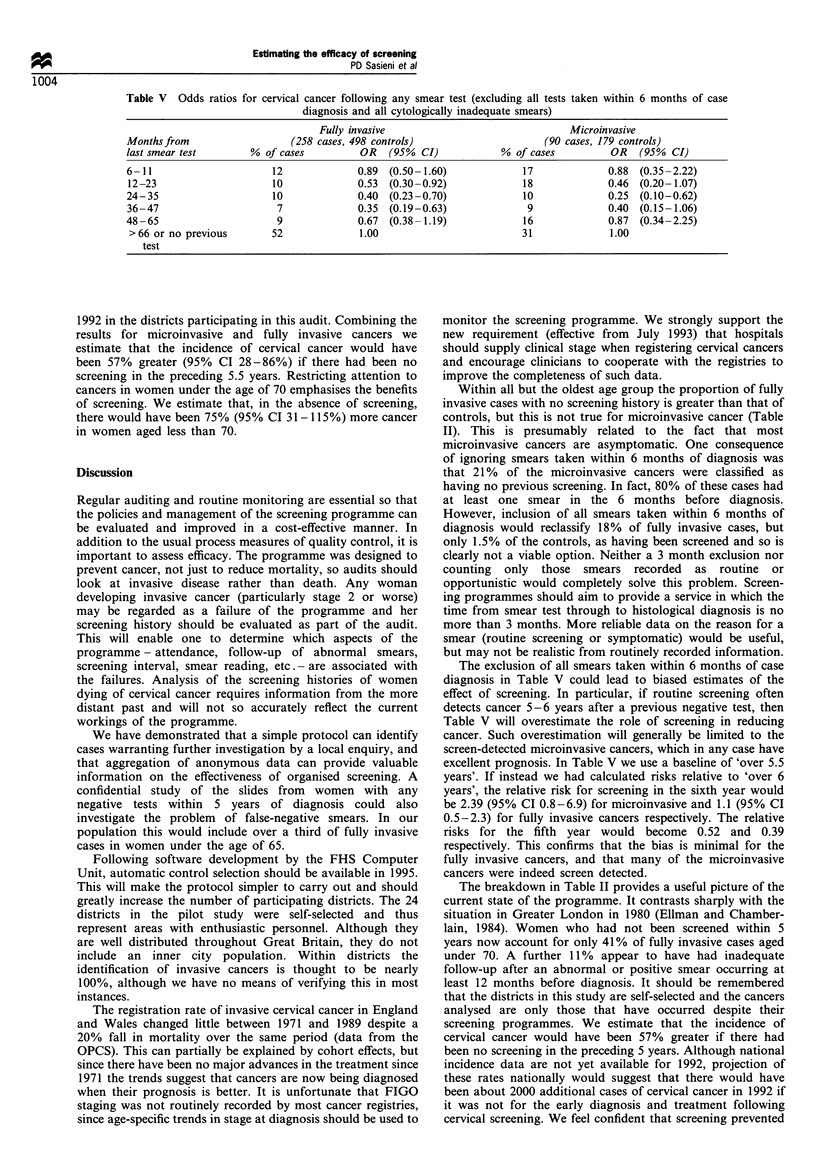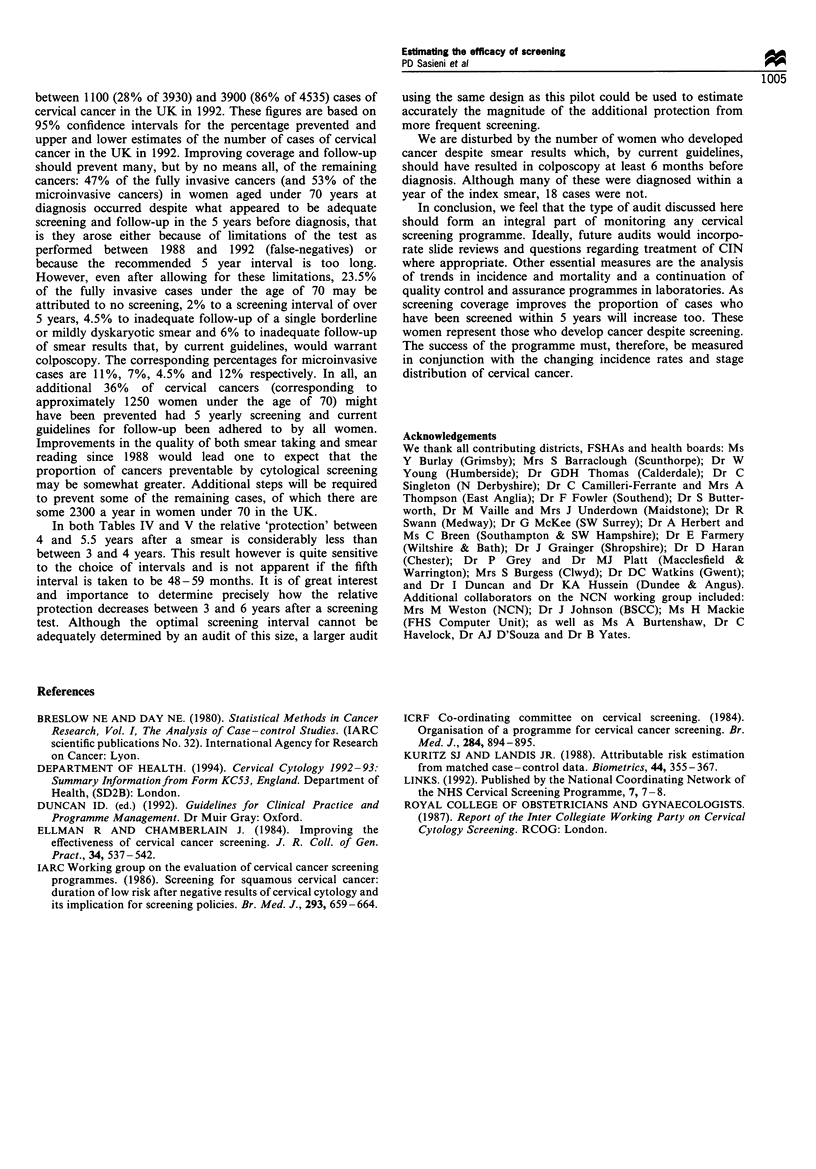# Estimating the efficacy of screening by auditing smear histories of women with and without cervical cancer. The National Co-ordinating Network for Cervical Screening Working Group.

**DOI:** 10.1038/bjc.1996.196

**Published:** 1996-04

**Authors:** P. D. Sasieni, J. Cuzick, E. Lynch-Farmery

**Affiliations:** Department of Mathematics, Statistics and Epidemiology, Imperial Cancer Research Fund, London, UK.

## Abstract

The screening histories of all 348 women with invasive cervical cancer diagnosed in 1992 in 24 self-selected district health authorities and health boards in England, Wales and Scotland were compared with those of 677 age- and residency-matched controls. The controls were randomly selected from the family health services authority (FHSA) register. Screening histories, comprising the dates and results of all smears taken before the date of diagnosis of the patient's cancer, were determined from the FHSA computer and laboratory records. We estimate that the number of cases of cervical cancer in participating districts in 1992 would have been 57% (95% confidence interval 28-86%) greater if there had been no previous screening. In women under the age of 70 it would have been approximately 75% (31-115%) greater. Extrapolation of the results from this pilot suggests that screening prevented between 1100 and 3900 cases of invasive cervical cancer in the UK in 1992. Women with stage 1B cancer or worse were more likely to have no record of previous screening than controls: 47% of these women under the age of 70 had been adequately screened according to current (5 yearly screening) guidelines, compared with 75% of matched controls. Thirteen per cent of all patients under age 70 had screening histories indicative of inadequate follow-up of smears requiring colposcopy. The proportion of microinvasive cases with screening predating diagnosis was similar to the proportion of controls. There was a strong correlation between stage and age: 56% of cancers in women under 35 were microinvasive compared with just 9% in women 65 years or over. The 'relative protection' following a negative smear was greatest in the first 12 months and fell off towards the end of the fifth year. These data suggest that full adherence to current guidelines could perhaps have prevented another 1250 cases, but additional steps would have been required to prevent some of the 2300 remaining cases in women under the age of 70.


British Journal of Cancer (1996) 73, 1001-1005
© 1996 Stockton Press All rights reserved 0007-0920/96 $12.00

### Estimating the efficacy of screening by auditing smear histories of women with and without cervical cancer

PD Sasieni[1], J Cuzick[1], E Lynch-Farmery[2] and The National Co-ordinating Network for Cervical Screening Working Group

[1]*Department of Mathematics, Statistics and Epidemiology, Imperial Cancer Research Fund, PO Box 123, Lincoln's Inn Fields, London WC2A 3PX;* [2]*Directorate of Public Health, Wiltshire and Bath Health Authority, Southgate House, Pans Lane, Devizes SN10 5EQ, UK.*

**Summary** The screening histories of all 348 women with invasive cervical cancer diagnosed in 1992 in 24 self-selected district health authorities and health boards in England, Wales and Scotland were compared with those of 677 age- and residency-matched controls. The controls were randomly selected from the family health services authority (FHSA) register. Screening histories, comprising the dates and results of all smears taken before the date of diagnosis of the patient's cancer, were determined from the FHSA computer and laboratory records. We estimate that the number of cases of cervical cancer in participating districts in 1992 would have been 57% (95% confidence interval 28-86%) greater if there had been no previous screening. In women under the age of 70 it would have been approximately 75% (31-115%) greater. Extrapolation of the results from this pilot suggests that screening prevented between 1100 and 3900 cases of invasive cervical cancer in the UK in 1992. Women with stage 1B cancer or worse were more likely to have no record of previous screening than controls: 47% of these women under the age of 70 had been adequately screened according to current (5 yearly screening) guidelines, compared with 75% of matched controls. Thirteen per cent of all patients under age 70 had screening histories indicative of inadequate follow-up of smears requiring colposcopy. The proportion of microinvasive cases with screening predating diagnosis was similar to the proportion of controls. There was a strong correlation between stage and age: 56% of cancers in women under 35 were microinvasive compared with just 9% in women 65 years or over. The 'relative protection' following a negative smear was greatest in the first 12 months and fell off towards the end of the fifth year. These data suggest that full adherence to current guidelines could perhaps have prevented another 1250 cases, but additional steps would have been required to prevent some of the 2300 remaining cases in women under the age of 70.

**Keywords:** audit; cervical screening; Pap smear; screening

Mortality from cervical cancer in the UK has fallen by over 30% since the introduction of screening in the 1960s. However, much of this is because of falling rates in older women and could be a cohort effect unrelated to screening. The need for an effectively managed national programme was realised by the mid-1980s (ICRF, 1984; RCOG, 1987). This led to the introduction of a computerised call and recall system for women aged between 20 and 64, central to the current programme, in 1988. The invitation-based system, together with target payments for general practitioners, has improved coverage from between 40% and 60% in 1989 to 80% in 1992 and 83% in 1993 (Department of Health, 1994). Considerable effort has gone into improving smear taking and reading, and the follow-up of women with abnormal results since 1988.

Despite the improved coverage and management, it is necessary to examine whether screening is achieving its goal of reducing the incidence of cervical cancer and identify areas of current practice for improvement. In 1991 we proposed a procedure for regular auditing of the NHS Cervical Screening Programme using routinely collected data (LINKS, 1992). The design was purposely kept simple and only essential data were collected. Such an audit could also serve as the first step of a more in-depth enquiry that may be required from time to time. This paper reports on the pilot phase of a National Co-ordinating Network project in which 24 districts from England, Wales and Scotland participated and covers cancers diagnosed in 1992. The aim of the pilot was to study the feasibility of using a common protocol in multiple districts to audit local programmes and to provide important up-to-date information on the performance of the national programme by pooling core data. The pooled data could be used to determine the importance of coverage, screening interval and follow-up of abnormal smears in preventing invasive disease. Further calculations yield estimates of the proportion of cancers that have been prevented by the current screening programme and the potential for further reductions associated with improvements in these aspects of the programme. We are thus able to assess the effectiveness of cervical screening despite the absence of randomised controlled trials. The local data can then be used to identify priorities for future local spending. Data presented here are from the pilot only and the estimates obtained should be regraded as preliminary. They indicate the sort of results that would be available if the protocol was routinely used in all districts. Nevertheless, we believe that the estimates presented here are the best available for the UK screening programme.

### Materials and methods

Following group discussions a protocol was adopted and publicised through the National Co-ordinating Network for Cervical Screening (LINKS, 1992). The 24 self-selected districts included in the pilot come from 9 of the 14 English regions (1992 boundaries) plus Wales and Scotland. Lists of all cases of invasive, including microinvasive, cervical cancer were obtained from local pathology laboratories. Local coordinators recorded the date of diagnosis, stage and histology together with date of birth and certain identifying details such as name and address of each case. The coordinators sought two age-matched controls per case from the computerised registry held by the local family health services authority (FHSA). This contains a list of all people registered with a local general practitioner (GP) together with certain information including details of cervical smears. Since women who have had a hysterectomy are excluded from routine cervical screening, this information

Correspondence: PD Sasieni
Received 30 May 1995; revised 9 November 1995; accepted 9 November 1995

should also be recorded on the FHSA computer. One control was registered with the same GP as the case and the other, though from the same district, was registered with a different group practice. Controls with no screening history were included. Women known to have had a hysterectomy were excluded. The GP control was intended to give a partial match for social class. We realised that this might lead to overmatching since coverage is influenced by the enthusiasm of the individual GP, not just by the socioeconomic mix of the population. For this reason a second control from a different group practice was used. One district only sought GP controls, so there are not quite two controls per case overall. Screening histories were obtained from records held on the FHSA computer and from cytology laboratories. No attempt was made to obtain more detailed or complete data from GP notes. Anonymised data were sent to one of the authors (PDS) for collation and analysis. Districts were encouraged to obtain information on treatment where relevant. Several found that the standard protocol revealed aspects of their programmes that could be improved and this was done, thus completing the audit cycle.

Microinvasive cases (FIGO stage 1A) are considered separately from other cases in most analyses. This is because they have excellent prognosis and may be regarded as partial successes of the screening programme, in that most would not have been diagnosed with such good prognosis in the absence of screening.

For the purpose of this study we defined a negative smear to be one with negative cytology (code 2) that did not immediately follow a previous borderline or dyskaryotic smear. (Thus the second consecutive smear with negative cytology following a borderline or worse test will be classified as negative). We also adopted a formal definition of cytological results that would lead to colposcopy. The following point system is based on current recommendations. Counting moderate or severe dyskaryosis as three points, mild dyskaryosis as two and borderline changes as one point, women should be referred for colposcopy when they have accumulated at least three points from their smear results: two consecutive negative smears wipes the slate clean.

Formal comparisons between cases and controls were made using conditional logistic regression (Breslow and Day, 1980). Population attributable risks were calculated using the techniques appropriate to matched case–control data (Kuritz and Landis, 1988). The attributable risks are presented as percentages of the actual rate of cervical cancer, rather than of the estimate of the rate that would have existed in the absence of screening. One analysis considers the time to the most recent adequate cervical smear excluding (in both cases and controls) any within 6 months of diagnosis of the case's cancer. In this way we attempt to discover whether diagnosis of cervical intraepithelial neoplasia (CIN) following positive cytology helps to prevent invasive cancer. We take the pragmatic view that where invasive cancer is diagnosed within 6 months of a non-negative smear, that smear may have resulted from symptoms. Inevitably, however, some will have been asymptomatic.

### Results

Of the 348 cases, 90 were known to be microinvasive (1A) and 186 were known to be stage 1B or worse. Of the remainder, 24 were stage 1 (not specified A or B) and 48 were of unknown stage. At least 72% of cancers in women aged under 50 were stage 1. There was a clear association between stage and age. Fifty-six per cent of cases under 35 years were definitely stage 1A, compared with 14% of those aged 65 to 74 years and none of those aged 75 or older. We use the term 'fully invasive' to refer to those invasive cancers not known to be microinvasive.

From Table I it can be seen that 45% of the fully invasive cases had no smears recorded other than possibly within 6 months of diagnosis. The comparative figures are 29% for controls ($P=0.002$) and 21% for microinvasive cancers. In this respect stage 1A cases were not significantly different from the controls ($P=0.27$). Coverage was strongly dependent on age, with a steady decline in coverage with increasing age. Cases were more likely to have no screening history than controls in all but the oldest age group (75+).

Table II considers compliance with screening guidelines formally introduced in 1992 (Duncan, 1992). Before this there were no accepted national guidelines. Since the screening programme invites women up to the age of 64, it could be argued that women aged 65–69 in 1992 should have been screened in the previous 5 years (i.e. between 6 months and 5.5 years before the date of diagnosis). Approximately half of the cases under the age of 70 appear to have been adequately screened and followed up within the previous 5 years. However a substantial proportion arose in women who had had a previous non-negative result which apparently was not adequately followed up by today's standards. Whereas fewer than 0.5% of controls had evidence of inadequate cytological follow-up, 5% of cases had a borderline or mildly dyskaryotic result with no cytological follow-up within the next 6 months. In a further 8% of cases, diagnosis occurred at least 6 months after a dyskaryotic smear warranting colposcopy. Some of these women will have developed cancer after unsuccessful treatment, but the routine nature of our data was not adequate to determine how many.

The remaining analyses concentrate on women aged 64 or less because few older women had any recorded screening.

**Table I** Percentage of women with no screening history up to 6 months before diagnosis

| *Age (years)* | *Microinvasive* | | *Fully invasive* | | *Controls* | |
|---|---|---|---|---|---|---|
| 20–34 | 3% | (1/35) | 14% | (5/36) | 9% | (12/139) |
| 35–49 | 21% | (7/34) | 29% | (22/77) | 13% | (28/215) |
| 50–64 | 50% | (7/14) | 43% | (27/63) | 26% | (40/154) |
| 65–74 | 57% | (4/7) | 68% | (36/53) | 60% | (68/113) |
| 75+ | | (0/0) | 90% | (26/29) | 91% | (51/56) |
| All ages | 21% | (19/90) | 45% | (116/258) | 29% | (199/677) |

**Table II** Compliance with recommended screening in women aged under 70 years, 6 months before diagnosis

| | *Microinvasive* (n=*89*) | | *Fully invasive* (n=*205*) | | *Controls* (n=*571*) | |
|---|---|---|---|---|---|---|
| Inadequate coverage | | | | | | |
| No history | 20% | 30% | 36% | 41% | 19% | 24% |
| No test in 5 year interval | 10% | | 6% | | 6% | |
| Inadequate follow-up | | | | | | |
| Of one abnormal test | 4% | 17% | 5% | 11% | 0.5% | 1% |
| After result requiring colposcopy | 12% | | 6% | | 0.5% | |
| Adequate screening history | 53% | | 47% | | 75% | |

Table III classifies screening histories according to a woman's worst smear ignoring those taken within six months of diagnosis. (A similar 6 month exclusion is applied to controls). For the microinvasive cancers, 18% had no previous screening history, whereas 39% had had an abnormal or positive test. In contrast, 31% of the other cases had no previous history and only 20% had a previous non-negative result. Disconcertingly, 48 women (18.5% of 259 aged under 65) developed invasive cancer within 3 years of a negative result: 19 were known to be stage 1B or worse. There was not a significant difference between the proportions of cases and controls with an abnormal or positive test followed by two consecutive negative smears (matched Mantel–Haenszel $\chi^2_1=2.9$, $P=0.09$). Eighteen (22%) microinvasive cases and 15 (8.5%) fully invasive cancers were diagnosed more than 6 months after a screening result requiring colposcopy (by our scoring system). In half of these women the time interval was over a year. A further 11 cases (four microinvasive) had no smears within 12 months of a single borderline or mildly dyskaryotic test. It is possible that some of these women had had colposcopy without our knowledge, but nevertheless this indicates a deficiency in the fail-safe system.

Table IV looks at the risk of developing fully invasive cervical cancer as a function of the number of years since a negative smear result (IARC, 1986). Apart from excluding microinvasive cancers, no attempt has been made to treat screen-detected cases differently from symptomatic ones. There is a general trend with time from last negative smear, with a relative protection of approximtely 5.6 (=1/0.18) in the first year decreasing to approximately 3.1 in the fourth year and 1.6 after between 48 and 65 months.

The percentage of women whose most recent negative test was within a given time interval is plotted in Figure 1 separately for controls, microinvasive and other cases. The figure shows that controls are far more likely than the cases to have had a recent negative test. Over the 4 years before case diagnosis, controls were screened at a steady rate of 16.5% per year. As expected, stage 1A cases were extremely unlikely to have had a negative result just before diagnosis (since most are screen detected), but the cumulative percentage of microinvasive cases with a negative result becomes indistinguishable from that of the other cases after 3 or 4 years. The figure also shows that very few controls had their first negative smear between 5 and 6 years before inclusion in the study, but that this was quite common among cases. This presumably reflects women whose cancers were diagnosed after a routine screening test 5 years after a previous negative result.

Analyses that focus on the period of low risk following a negative test will overestimate the reduction in risk due to screening overall because women with abnormal or positive smears may go on to develop cancer. To study the protection given by participation in the screening programme, we also considered the time to the last adequate smear (i.e. one that was not classified as inadequate on cytology) excluding all those within 6 months of diagnosis. Table V shows the estimated relative risks of developing cervical cancer more than 6 months after any smear test. It is apparent from the table that the risk of disease is greater in the first year (months 7–12) than in years 2–5. This reflects those cancers that are not diagnosed within 6 months of an initial positive or abnormal test. Data in Tables I and V can be used to estimate the proportion of cervical cancer that has been prevented by screening in the five years between 1988 and

**Table IV** Odds ratios for cervical cancer[a] by time elapsed since lase negative[b] smear based on all 258 fully invasive cases and their 498 matched controls

| *Months since last negative smear* | *% of cases* | *OR* | *(95% CI)* |
|---|---|---|---|
| 0–11 | 6 | 0.18 | (0.09–0.35) |
| 12–23 | 8 | 0.33 | (0.18–0.61) |
| 24–35 | 9 | 0.26 | (0.14–0.47) |
| 36–47 | 7 | 0.32 | (0.17–0.56) |
| 48–65 | 11 | 0.64 | (0.36–1.14) |
| >66 or no previous test | 60 | 1.00 | |

[a] Excluding those cases known to be microinvasive. [b] Excludes negative tests that immediately follow a non-negative test.

**Figure 1** Distribution of the time since the most recent negative smear test in cases and controls. Women are only included while they are aged between 20 and 64 years.

**Table III** Breakdown of screening histories of women under 65 years old up to 6 months before diagnosis

| *Screening history* | *Microinvasive* | *Fully invasive* | *Controls* |
|---|---|---|---|
| No history | 15 (18%) | 54 (31%) | 80 (15%) |
| All negative | | | |
| Most recent within | | | |
| 3 years | 9 (11%) | 39 (22%) | 241 (48%) |
| 4–5 years | 12 (14%) | 25 (14%) | 118 (23%) |
| over 5 years | 15 (18%) | 22 (13%) | 41 (8%) |
| One borderline or mild | | | |
| Followed by two negatives | 3 (4%) | 3 (2%) | 10 (2%) |
| Diganosis over 6 months later | 7 (8%) | 11 (6%) | 5 (1%) |
| Cytoloty warrantng colposcopy | | | |
| Followed by two negatives | 4 (5%) | 7 (4%) | 11 (2%) |
| Diagnosis over 6 months later | 18 (22%) | 15 (9%) | 2 (<1%) |
| Total | 83 (100%) | 176 (100%) | 506 (100%) |

Table V Odds ratios for cervical cancer following any smear test (excluding all tests taken within 6 months of case diagnosis and all cytologically inadequate smears)

| Months from last smear test | Fully invasive (258 cases, 498 controls) % of cases | OR | (95% CI) | Microinvasive (90 cases, 179 controls) % of cases | OR | (95% CI) |
|---|---|---|---|---|---|---|
| 6–11 | 12 | 0.89 | (0.50–1.60) | 17 | 0.88 | (0.35–2.22) |
| 12–23 | 10 | 0.53 | (0.30–0.92) | 18 | 0.46 | (0.20–1.07) |
| 24–35 | 10 | 0.40 | (0.23–0.70) | 10 | 0.25 | (0.10–0.62) |
| 36–47 | 7 | 0.35 | (0.19–0.63) | 9 | 0.40 | (0.15–1.06) |
| 48–65 | 9 | 0.67 | (0.38–1.19) | 16 | 0.87 | (0.34–2.25) |
| > 66 or no previous test | 52 | 1.00 | | 31 | 1.00 | |

1992 in the districts participating in this audit. Combining the results for microinvasive and fully invasive cancers we estimate that the incidence of cervical cancer would have been 57% greater (95% CI 28–86%) if there had been no screening in the preceding 5.5 years. Restricting attention to cancers in women under the age of 70 emphasises the benefits of screening. We estimate that, in the absence of screening, there would have been 75% (95% CI 31–115%) more cancer in women aged less than 70.

### Discussion

Regular auditing and routine monitoring are essential so that the policies and management of the screening programme can be evaluated and improved in a cost-effective manner. In addition to the usual process measures of quality control, it is important to assess efficacy. The programme was designed to prevent cancer, not just to reduce mortality, so audits should look at invasive disease rather than death. Any woman developing invasive cancer (particularly stage 2 or worse) may be regarded as a failure of the programme and her screening history should be evaluated as part of the audit. This will enable one to determine which aspects of the programme – attendance, follow-up of abnormal smears, screening interval, smear reading, etc. – are associated with the failures. Analysis of the screening histories of women dying of cervical cancer requires information from the more distant past and will not so accurately reflect the current workings of the programme.

We have demonstrated that a simple protocol can identify cases warranting further investigation by a local enquiry, and that aggregation of anonymous data can provide valuable information on the effectiveness of organised screening. A confidential study of the slides from women with any negative tests within 5 years of diagnosis could also investigate the problem of false-negative smears. In our population this would include over a third of fully invasive cases in women under the age of 65.

Following software development by the FHS Computer Unit, automatic control selection should be available in 1995. This will make the protocol simpler to carry out and should greatly increase the number of participating districts. The 24 districts in the pilot study were self-selected and thus represent areas with enthusiastic personnel. Although they are well distributed throughout Great Britain, they do not include an inner city population. Within districts the identification of invasive cancers is thought to be nearly 100%, although we have no means of verifying this in most instances.

The registration rate of invasive cervical cancer in England and Wales changed little between 1971 and 1989 despite a 20% fall in mortality over the same period (data from the OPCS). This can partially be explained by cohort effects, but since there have been no major advances in the treatment since 1971 the trends suggest that cancers are now being diagnosed when their prognosis is better. It is unfortunate that FIGO staging was not routinely recorded by most cancer registries, since age-specific trends in stage at diagnosis should be used to monitor the screening programme. We strongly support the new requirement (effective from July 1993) that hospitals should supply clinical stage when registering cervical cancers and encourage clinicians to cooperate with the registries to improve the completeness of such data.

Within all but the oldest age group the proportion of fully invasive cases with no screening history is greater than that of controls, but this is not true for microinvasive cancer (Table II). This is presumably related to the fact that most microinvasive cancers are asymptomatic. One consequence of ignoring smears taken within 6 months of diagnosis was that 21% of the microinvasive cancers were classified as having no previous screening. In fact, 80% of these cases had at least one smear in the 6 months before diagnosis. However, inclusion of all smears taken within 6 months of diagnosis would reclassify 18% of fully invasive cases, but only 1.5% of the controls, as having been screened and so is clearly not a viable option. Neither a 3 month exclusion nor counting only those smears recorded as routine or opportunistic would completely solve this problem. Screening programmes should aim to provide a service in which the time from smear test through to histological diagnosis is no more than 3 months. More reliable data on the reason for a smear (routine screening or symptomatic) would be useful, but may not be realistic from routinely recorded information.

The exclusion of all smears taken within 6 months of case diagnosis in Table V could lead to biased estimates of the effect of screening. In particular, if routine screening often detects cancer 5–6 years after a previous negative test, then Table V will overestimate the role of screening in reducing cancer. Such overestimation will generally be limited to the screen-detected microinvasive cancers, which in any case have excellent prognosis. In Table V we use a baseline of 'over 5.5 years'. If instead we had calculated risks relative to 'over 6 years', the relative risk for screening in the sixth year would be 2.39 (95% CI 0.8–6.9) for microinvasive and 1.1 (95% CI 0.5–2.3) for fully invasive cancers respectively. The relative risks for the fifth year would become 0.52 and 0.39 respectively. This confirms that the bias is minimal for the fully invasive cancers, and that many of the microinvasive cancers were indeed screen detected.

The breakdown in Table II provides a useful picture of the current state of the programme. It contrasts sharply with the situation in Greater London in 1980 (Ellman and Chamberlain, 1984). Women who had not been screened within 5 years now account for only 41% of fully invasive cases aged under 70. A further 11% appear to have had inadequate follow-up after an abnormal or positive smear occurring at least 12 months before diagnosis. It should be remembered that the districts in this study are self-selected and the cancers analysed are only those that have occurred despite their screening programmes. We estimate that the incidence of cervical cancer would have been 57% greater if there had been no screening in the preceding 5 years. Although national incidence data are not yet available for 1992, projection of these rates nationally would suggest that there would have been about 2000 additional cases of cervical cancer in 1992 if it was not for the early diagnosis and treatment following cervical screening. We feel confident that screening prevented between 1100 (28% of 3930) and 3900 (86% of 4535) cases of cervical cancer in the UK in 1992. These figures are based on 95% confidence intervals for the percentage prevented and upper and lower estimates of the number of cases of cervical cancer in the UK in 1992. Improving coverage and follow-up should prevent many, but by no means all, of the remaining cancers: 47% of the fully invasive cancers (and 53% of the microinvasive cancers) in women aged under 70 years at diagnosis occurred despite what appeared to be adequate screening and follow-up in the 5 years before diagnosis, that is they arose either because of limitations of the test as performed between 1988 and 1992 (false-negatives) or because the recommended 5 year interval is too long. However, even after allowing for these limitations, 23.5% of the fully invasive cases under the age of 70 may be attributed to no screening, 2% to a screening interval of over 5 years, 4.5% to inadequate follow-up of a single borderline or mildly dyskaryotic smear and 6% to inadequate follow-up of smear results that, by current guidelines, would warrant colposcopy. The corresponding percentages for microinvasive cases are 11%, 7%, 4.5% and 12% respectively. In all, an additional 36% of cervical cancers (corresponding to approximately 1250 women under the age of 70) might have been prevented had 5 yearly screening and current guidelines for follow-up been adhered to by all women. Improvements in the quality of both smear taking and smear reading since 1988 would lead one to expect that the proportion of cancers preventable by cytological screening may be somewhat greater. Additional steps will be required to prevent some of the remaining cases, of which there are some 2300 a year in women under 70 in the UK.

In both Tables IV and V the relative 'protection' between 4 and 5.5 years after a smear is considerably less than between 3 and 4 years. This result however is quite sensitive to the choice of intervals and is not apparent if the fifth interval is taken to be 48–59 months. It is of great interest and importance to determine precisely how the relative protection decreases between 3 and 6 years after a screening test. Although the optimal screening interval cannot be adequately determined by an audit of this size, a larger audit using the same design as this pilot could be used to estimate accurately the magnitude of the additional protection from more frequent screening.

We are disturbed by the number of women who developed cancer despite smear results which, by current guidelines, should have resulted in colposcopy at least 6 months before diagnosis. Although many of these were diagnosed within a year of the index smear, 18 cases were not.

In conclusion, we feel that the type of audit discussed here should form an integral part of monitoring any cervical screening programme. Ideally, future audits would incorporate slide reviews and questions regarding treatment of CIN where appropriate. Other essential measures are the analysis of trends in incidence and mortality and a continuation of quality control and assurance programmes in laboratories. As screening coverage improves the proportion of cases who have been screened within 5 years will increase too. These women represent those who develop cancer despite screening. The success of the programme must, therefore, be measured in conjunction with the changing incidence rates and stage distribution of cervical cancer.

**Acknowledgements**

We thank all contributing districts, FSHAs and health boards: Ms Y Burlay (Grimsby); Mrs S Barraclough (Scunthorpe); Dr W Young (Humberside); Dr GDH Thomas (Calderdale); Dr C Singleton (N Derbyshire); Dr C Camilleri-Ferrante and Mrs A Thompson (East Anglia); Dr F Fowler (Southend); Dr S Butterworth, Dr M Vaille and Mrs J Underdown (Maidstone); Dr R Swann (Medway); Dr G McKee (SW Surrey); Dr A Herbert and Ms C Breen (Southampton & SW Hampshire); Dr E Farmery (Wiltshire & Bath); Dr J Grainger (Shropshire); Dr D Haran (Chester); Dr P Grey and Dr MJ Platt (Macclesfield & Warrington); Mrs S Burgess (Clwyd); Dr DC Watkins (Gwent); and Dr I Duncan and Dr KA Hussein (Dundee & Angus). Additional collaborators on the NCN working group included: Mrs M Weston (NCN); Dr J Johnson (BSCC); Ms H Mackie (FHS Computer Unit); as well as Ms A Burtenshaw, Dr C Havelock, Dr AJ D'Souza and Dr B Yates.

### References

BRESLOW NE AND DAY NE. (1980). *Statistical Methods in Cancer Research, Vol. I, The Analysis of Case–control Studies.* (IARC scientific publications No. 32). International Agency for Research on Cancer: Lyon.

DEPARTMENT OF HEALTH. (1994). *Cervical Cytology 1992–93: Summary Information from Form KC53, England.* Department of Health, (SD2B): London.

DUNCAN ID. (ed.) (1992). *Guidelines for Clinical Practice and Programme Management.* Dr Muir Gray: Oxford.

ELLMAN R AND CHAMBERLAIN J. (1984). Improving the effectiveness of cervical cancer screening. *J. R. Coll. of Gen. Pract.*, **34**, 537–542.

IARC Working group on the evaluation of cervical cancer screening programmes. (1986). Screening for squamous cervical cancer: duration of low risk after negative results of cervical cytology and its implication for screening policies. *Br. Med. J.*, **293**, 659–664.

ICRF Co-ordinating committee on cervical screening. (1984). Organisation of a programme for cervical cancer screening. *Br. Med. J.*, **284**, 894–895.

KURITZ SJ AND LANDIS JR. (1988). Attributable risk estimation from matched case–control data. *Biometrics*, **44**, 355–367.

LINKS. (1992). Published by the National Coordinating Network of the NHS Cervical Screening Programme, **7**, 7–8.

ROYAL COLLEGE OF OBSTETRICIANS AND GYNAECOLOGISTS. (1987). *Report of the Inter Collegiate Working Party on Cervical Cytology Screening.* RCOG: London.